# Human adenoviral type 54 keratoconjunctivitis accompanied by stellate keratitis and keratic precipitates: two cases

**DOI:** 10.1186/s12886-018-1025-6

**Published:** 2019-01-07

**Authors:** Kazuki Matsuura, Yuki Terasaka, Eiichi Uchio, Yusuke Saeki, Tsuguto Fujimoto, Nozomu Hanaoka, Dai Miyazaki, Yoshitsugu Inoue

**Affiliations:** 1 Department of Ophthalmology, Nojima Hospital, 2714-1 Sesaki-machi Kurayoshi City, Tottori, 682-0863 Japan; 2 Department of Ophthalmology, Faculty of Medicine, Fukuoka University, 45-1 7 chome Nanakuma Jonan-ku, Fukuoka City, Fukuoka Japan; 3 Infectious Diseases Surveillance Centre, National Institute of Infectious Diseases, 1-23-1 Toyama, Shinjuku-ku, Tokyo, Japan; 4 Department of Ophthalmology, Faculty of Medicine, Tottori University, 36-1 Nishicho Yonago City, Tottori, Japan

**Keywords:** Human adenoviral type 54, Keratoconjunctivitis, Stellate keratitis, Keratic precipitates

## Abstract

**Background:**

Of the 10 patients with adenoviral type 54 keratoconjunctivitis examined at Nojima Hospital, 2 developed stellate keratitis and mutton-fat keratic precipitates (KPs) following acute symptoms.

**Case presentation:**

We encountered 10 cases of epidemic keratoconjunctivitis from August to October 2017. All patients were adults with a mean age of 60.9 ± 10.0 years. The species D human adenovirus (HAdV)-54 was detected in the conjunctival scrapings of these patients. Fluorometholone instillation was administered during the first week for acute symptomatic relief.

Case 1: A 64-year-old female was prescribed with fluorometholone instillation, which was discontinued after 1 week when her symptoms alleviated. One week after discontinuation of the instillation, she presented with blurred vision in her left eye with KPs and multiple stellate keratitis. The anterior chamber had no apparent cells. Her symptoms disappeared after 1 week of betamethasone instillation.

Case 2: A 66-year-old female was prescribed with 0.1% fluorometholone instillation, which was discontinued within10 days. Three months after the appearance of initial symptoms, multiple subepithelial corneal infiltrates (MSI) appeared in her eyes. Stellate keratitis and dark-brown pigmentation were observed in the centres of MSI, with several cells in the anterior chamber. Betamethasone was prescribed, and MSI and stellate keratitis improved within 1 week. However, KPs were observed in the left eye. The instillation was continued for 3 more weeks until symptoms improved.

**Conclusions:**

MSI is an immune reaction that occurs after the disappearance of acute symptoms. Here, corneal findings and KPs were observed after improvement in eye redness and discontinuation of steroids. These symptoms were presumed to be secondary inflammation due to immune response to the adenoviral antigen.

The clinical features of HAdV-54 keratoconjunctivitis on the ocular surface are initially moderate, but become active in the subacute to chronic phases. This may develop atypical findings, including stellate keratitis with KPs. Although early steroid administration can relieve acute symptoms, it may facilitate chronic corneal immunological reaction.

## Background

Epidemic keratoconjunctivitis (EKC) is most commonly caused by species D human adenoviruses (HAdV), including types HAdV-8, HAdV-37, HAdV-56, HAdV-64 and HAdV-85 [1–4]. In Japan, the number of EKC cases caused by HAdV-8—the most common adenovirus worldwide [5]—has decreased, whereas the number of EKC cases caused by HAdV-37 and HAdV-54 has increased [1–3]. However, because antibodies of HAdV-54 show cross-neuralisation with those of HAdV-8, HAdV-54 may have been inadvertently included in previous reports on HAdV-8 surveillance [1, 2].

The occurrence of superficial punctate keratitis (SPK) during the acute phase of adenoviral keratoconjunctivitis and that of multiple subepithelial corneal infiltrates (MSI) during the subacute and chronic phases have been well reported. Clinical features are typically moderate in the early phase of HAdV-54 keratoconjunctivitis; however, the occurrence rate of MSI was higher than that in previous epidemics of several HAdV types [6, 7]. Uemura et al. reported that the clinical severity of HAdV-54 keratoconjunctivitis was mild, moderate and severe in 3.6, 94.6 and 1.8% of cases, respectively. No cases exhibited a conjunctival pseudomembrane, and corneal involvement was observed in only 1 case as SPK (1.8%) [6].

Akiyoshi et al. reported that the clinical presentation in the early stage of HAdV-54 infection resembles that of acute allergic keratoconjunctivitis and that the typical features of severe EKC subsequently appear [7]. Of 10 patients with adenoviral keratoconjunctivitis examined at Nojima Hospital, Japan, 2 developed stellate keratitis resembling Thygeson’s SPK with mutton-fat keratic precipitates (KPs), following improvement of acute symptoms. In this report, we analysed the clinical characteristics of HAdV-54 keratoconjunctivitis cases treated at our hospital and described the unusual findings.

## Case presentation

We encountered 10 cases of patients with EKCs at Nojima Hospital from August to October 2017. All patients were adults, and the mean age was 60.9 ± 10.0 years. Most patients visited the hospital within a few days of symptom onset (Table 1). Immunochromatography (Adenocheck, Santen, Osaka, Japan) was performed for diagnosis, and HAdV-54 was detected in the conjunctival scrapings of the patients using polymerase chain reaction amplification, sequencing and phylogenetic analysis, as previously reported [8]. For acute symptomatic relief, fluorometholone instillation was administered to all patients during the first week.

**Table 1:** Eight laboratory confirmed cases of epidemic keratoconjunctivitis

Case	Age (range)	Gender	Symptom	Date of onset	From onset to first visit (day)	Pseudo-membrane	KP and stellate keratitis	Subacute MSI (<3wks)	Chronic MSI (> 3 months)	Genotype
1	60~69	f	Unilateral	9–19–2017	3	–	+	–	–	54
2	60~69	f	Bilateral	9-22-2017	3	–	+	–	+	54
3	30~39	m	Unilateral	9-22-2017	3	–	–	+	+	54
4	40~49	f	Unilateral	9-22-2017	2	–	–	+	+	54
5	60~69	m	Bilateral	9-20-2017	2	–	–	–	–	54
6	70~79	f	Bilateral	9-16-2017	5	–	–	–	–	54
7	60~69	m	Unilateral	10-1-2017	1	–	–	–	–	54
8	60~69	m	Bilateral	9-26-2017	2	–	–	–	–	54
9	60~69	m	Unilateral	10–16-2017	2	–	–	–	–	54
10	60~69	m	Unilateral	10-23-2017	1	+	–	–	–	54

No patient had a history of immune or inflammatory disease generalised or localised to the eyes. The study protocol conformed to the tenets of the Declaration of Helsinki and was approved by the Ethics Review Committee of Nojima Hospital. Written informed consent was obtained from all patients.

**Fig 1:**
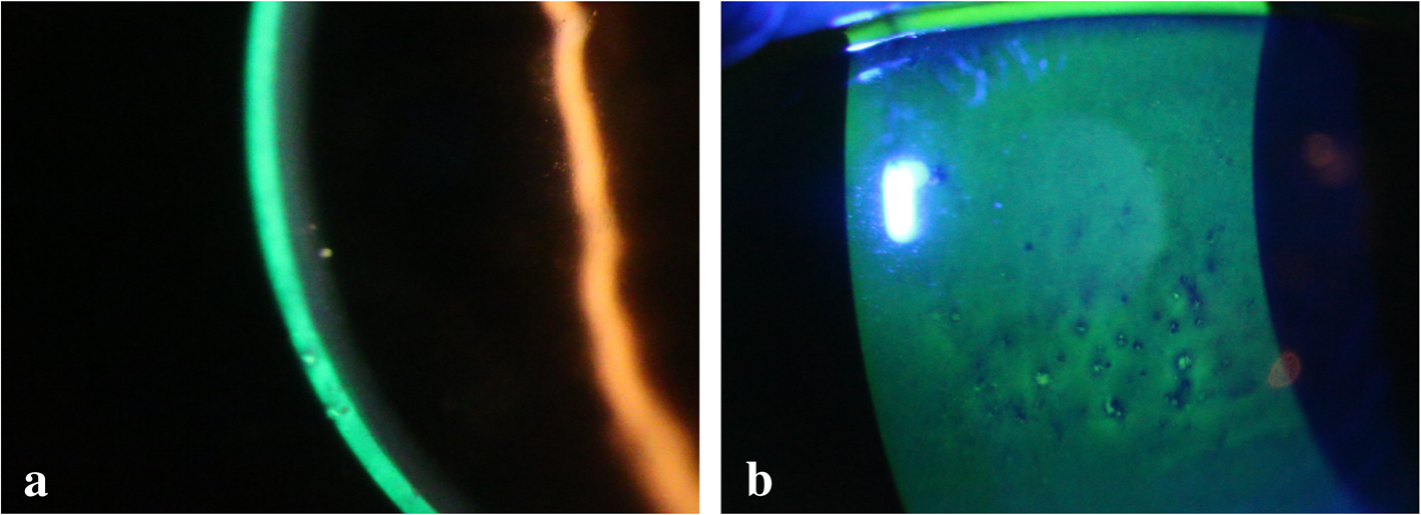
**a**. Mutton-fat KPs were observed. **b**. Stellate keratitis resembling Thygeson’s superficial punctate keratitis

Case 1: A 64-year-old female presented to our hospital in late September 2017 with severe redness and discharge in her left eye. Immunochromatography revealed that her conjunctival scrapings were positive for adenovirus. She was prescribed with levofloxacin and fluorometholone instillation 4 times daily, which was discontinued after 1 week (14 days from symptom onset) because her symptoms alleviated. However, 1 week after discontinuation she presented with blurred vision in her left eye. Examination revealed a visual acuity of 10/20 in the left eye with mutton-fat KPs and multiple stellate keratitis (Fig. 1). The anterior chamber had no apparent cells or flare. She was subsequently prescribed with levofloxacin and betamethasone 4 times daily in the left eye. The mutton-fat KPs and stellate keratitis disappeared after 1 week, and visual acuity recovered to 20/20.

Case 2: A 66-year-old female presented to our hospital in late September 2017 with redness in both eyes. Immunochromatography tests were positive for adenovirus. The patient was prescribed with 0.1% fluorometholone instillation 4 times daily, which was discontinued after 10 days when inflammation improved.

**Fig 2:**
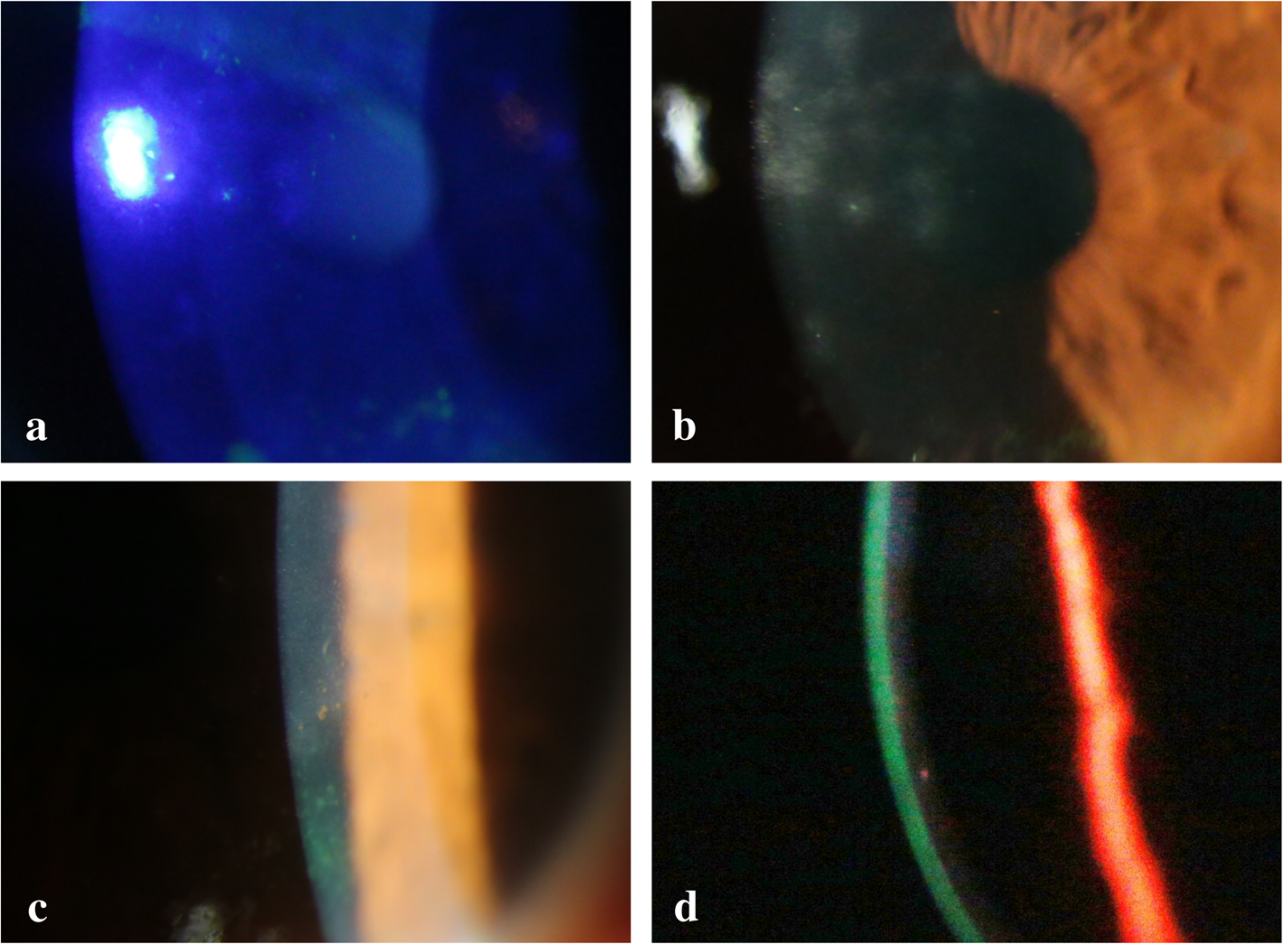
**a**, **b**. The centres of some MSI-stained coarsely. **c**. Dark-brown pigmentation was observed in the centres of MSIs. **d**. After 1 week, mutton-fat KPs were observed

Three months (98 days) after the initial symptoms, she presented with MSI with a foreign body sensation and blurred vision in both eyes (visual acuity, 20/25 in each eye). Examination revealed stellate keratitis-like fluorescein staining and dark-brown pigmentation in the centres of MSI with a few cells in the anterior chamber (Figs. 2a, b, c). The patient was prescribed with betamethasone instillation 4 times daily in her left eye.

MSI and stellate keratitis improved within 1 week; however, mutton-fat KPs were observed in the left eye (Fig. 2d). The betamethasone instillations were continued for 3 more weeks until the symptoms improved.

After healing, the second steroid instillation was gradually reduced over a period of 6 and 8 weeks in cases 1 and 2, respectively. Unpleasant symptoms, such as photophobia or blurred vision, were not observed over an 8-month observation period.

## Discussion and conclusions

Uveitis is a rare finding and has been described only in patients with severe keratitis in adenoviral keratoconjunctivitis [9, 10]. Two cases of KP with severe stromal keratitis with HAdV-19 and HAdV-37 appearing 2–3 weeks after the onset of conjunctivitis have been reported [9].

A study analysing the symptoms of different adenoviral serotypes showed that HAdV-8 was characteristic. SPK in HAdV-8 infection leads to coarse SPK with a slightly delayed onset (mean, over 3 weeks) and can progress into subepithelial lesions. Of the 21 patients in the study, 3 presented with mild uveitis, 3 with coarse SPK and 2 with diffuse stromal keratitis [10].

In the cases presented here, corneal stromal oedema was not observed. However, mild uveitis appeared after the symptoms of conjunctivitis alleviated and showed similar progression. Although it is unclear whether SPK coarseness described in the aforementioned study is similar to the stellate keratitis observed in the present cases, the consistency in their onset times suggests a possible similarity.

Diffuse SPK is often accompanied with acute adenoviral keratoconjunctivitis. However, in the cases presented here, epithelial damage appeared after improvement of acute symptoms. In case 1, multiple stellate keratitis was observed without MSI. In case 2, stellate keratitis-like staining was observed in the centre of several MSI lesions and pigmentation was also observed in the centres of several MSI lesions. This suggests that stellate keratitis in case 1 was either a precursory symptom or a mild finding of MSI. In case 2, stellate keratitis was presumed to have progressed into MSI with the healing process causing pigmentation. The site around the stellate keratitis-like staining appeared slightly elevated, and a lack of fluorescein staining resulted in a focal dry up (Fig. 1). Thus, the dark-brown pigmentation may have resulted from iron deposition in the form of hemosiderin, similar to Fleischer ring formation in keratoconus and Hudson–Stahli line.

MSI is considered to be an immune reaction that occurs following the improvement of acute symptoms. In the present report, corneal findings and mutton-fat KPs were observed after improvement of eye redness and discontinuation of steroids. MSI also occurred in the chronic phase in 3 cases, including 2 with recurrence (Table 1). These symptoms were presumed to be secondary inflammation due to an immunological reaction to the adenoviral antigen.

Despite moderate presentation in the early phase of cases with HAdV-54, a high incidence of MSI (24 of 31 cases, 77%) was reported by Uemura et al [6] This suggests that the immunological reaction in the course of HAdV-54 keratoconjunctivitis is more active compared with other HAdV types, although its clinical features on the ocular surface are moderate in the early phase. In the present study, the incidence of MSI was not as high (3 of 10 patients, 30%), despite a relatively severe presentation, including 1 case with pseudomembrane. This difference may be related to our patients all being adults, whereas 32 of 55 patients (58.2%) and 7 of 13 (53.8%) patients were children in the reports by Uemueas and Akiyoshis, respectively [6, 7]. Severe keratitis in the early phase is possibly less likely to occur in young individuals.

Early administration of corticosteroids for adenoviral keratoconjunctivitis remains controversial because chronic adenoviral conjunctivitis is associated with corticosteroid use [11]. Aforementioned studies [6, 7, 9, 10] did not refer to the early administration of steroid instillation. Administration of corticosteroids decreases the inflammation and provides significant symptomatic relief. In contrast, corticosteroids enhance viral replication and increase the duration of viral shedding. These effects have been demonstrated even after a short period of corticosteroid use [12]. The incidence of MSI in the subacute phase may have been lower because of the early application of steroids, thereby elevating the risk of the occurrence or recurrence of MSI in the chronic phase.

The clinical features of HAdV-54 keratoconjunctivitis on the ocular surface may seem moderate in the early phase. In contrast, the immunological reaction in the subacute to chronic phases is more active leading to the development of atypical findings, including stellate keratitis and mutton-fat KPs. Although early administration of steroids can relieve acute symptoms, it may also facilitate chronic corneal immunological reactions.

## Abbreviation

EKC: Epidemic keratoconjunctivitis
HAdV: Species D human adenovirus
KPs: Keratic precipitates
MSI: Multiple subepithelial corneal infiltrates
SPK: Superficial punctate keratitis
